# Achieving dietary recommendations and reducing greenhouse gas emissions: modelling diets to minimise the change from current intakes

**DOI:** 10.1186/s12966-016-0370-1

**Published:** 2016-04-07

**Authors:** Graham W. Horgan, Amandine Perrin, Stephen Whybrow, Jennie I. Macdiarmid

**Affiliations:** Biomathematics and Statistics Scotland, Aberdeen, AB25 2ZD UK; Public Health Nutrition Research Group, Rowett Institute of Nutrition and Health, University of Aberdeen, Aberdeen, AB25 2ZD UK

**Keywords:** Dietary recommendations, Greenhouse gas emissions, Linear programming, Sustainable diets, Modelling

## Abstract

**Background:**

Average population dietary intakes do not reflect the wide diversity of dietary patterns across the population. It is recognised that most people in the UK do not meet dietary recommendations and have diets with a high environmental impact, but changing dietary habits has proved very difficult. The purpose of this study was to investigate the diversity in dietary changes needed to achieve a *healthy diet* and a healthy diet with lower greenhouse gas emissions (GHGE) (referred to as *a sustainable diet*) by taking into account each individual’s current diet and then minimising the changes they need to make.

**Methods:**

Linear programming was used to construct two new diets for each adult in the UK National Diet and Nutrition Survey (*n* = 1491) by minimising the changes to their current intake. Stepwise changes were applied until (i) dietary recommendations were achieved and (ii) dietary recommendations and a GHGE target were met. First, gradual changes (≤50 %) were made to the amount of any foods currently eaten. Second, new foods were added to the diet. Third, greater reductions (≤75 %) were made to the amount of any food currently eaten and finally, foods were removed from the diet.

**Results:**

One person out of 1491 in the sample met all the dietary requirements based on their reported dietary intake. Only 7.5 and 4.6 % of people achieved a healthy diet and a sustainable diet, respectively, by changing the amount of any food they currently ate by up to 50 %. The majority required changes to the amount of each food eaten plus the addition of new foods. Fewer than 5 % had to remove foods they ate to meet recommendations. Sodium proved the most difficult nutrient recommendation to meet. The healthy diets and sustainable diets produced a 15 and 27 % reduction in greenhouse gas emissions respectively.

**Conclusions:**

Since healthy diets alone do not produce substantial reductions in greenhouse gas emissions, dietary guidelines need to include recommendations for environmental sustainability. Minimising the shift from current dietary intakes is likely to make dietary change more realistic and achievable.

## Background

Healthy diets are based on dietary recommendations that combine minimum (e.g. vitamins, minerals, fibre) and maximum (e.g. total fat, saturated fat, sugar, sodium) nutrient intakes [1, 2], plus food-based guidelines for fruit & vegetables, fish, red & processed meat [3, 4]. In many countries the average dietary intake of the population fails to meet these recommendations, which is contributing to high levels of diet related non-communicable disease and obesity [5, 6]. Poor dietary habits place major health, social and economic burdens on societies, but attempts to improve dietary intakes and change behaviour have had limited success. Added to the health concerns is the environmental damaged associated with particular dietary patterns, for example the contribution to climate change of diets with high intakes of meat and dairy products, seen in many higher income countries [7, 8]. This has implications for global food security, which has re-stimulated the debate about the need for healthy and more environmentally sustainable diets. One environmental impact is the contribution of food production and dietary choices on climate change. It is estimated that globally the food system accounts for between 19 and 29 % of all greenhouse gas emissions (GHGE) [9]. While there is scope to reduce GHGE in the production of food, it is recognised now that this alone will not be sufficient to meet targets set for the reduction in GHGE, and therefore to achieve these targets dietary intakes will need to change [10, 11]. The dietary changes to reduce the environmental impact need to be identified and complement dietary changes required for a healthy diet. The greater challenge is how to encourage people to make these dietary changes and change current eating habits across the population.

Some countries have developed dietary guidelines for sustainable diets, which go beyond nutrition and include recommendations to limit environmental impacts, typically to reduce GHGE [12–14]. New dietary guidelines were published in The Netherlands in 2011 [12] and Sweden in 2015 [13], which incorporate environmental sustainability, such as limiting meat consumption and choosing sustainably produced fish. Environmental dietary recommendations typically focus on limiting consumption of animal based products since production of livestock tends to have the highest environmental impacts (e.g. GHGE, land and water usage [15, 16]). Although there is support for the concept of healthy and sustainable diets, changing dietary behaviour, whether to improve health (e.g. reduce high fat and high sugar foods) or reduce GHGE (e.g. fewer meat and dairy products), is often viewed as undesirable by the general public and challenging in the current food environment [17]. A limitation of many dietary guidelines is that they fail to take account of social aspects of eating and the reasons behind peoples’ food choices, such as habits, preferences, affordability, circumstance, culture and social norms [18]. Recent dietary guidelines produced by the Brazilian government are a welcome exception, where some of the cultural aspects of eating have been integrated into the recommendation [19]. Nutrient recommendations can be achieved with many different combinations of food and drinks, meaning that dietary changes can accommodate some of the variation in social drivers of individual food choices. Achieving and sustaining healthy dietary intakes in the population has proved difficult, so understanding the minimum changes individuals need to make to improve current dietary intakes may be a more effective approach.

Previous research has modelled diets to meet dietary recommendations for health and lower GHGE, taking the average population dietary intake as the baseline [20–22]. These studies have identified general changes needed to population dietary intakes but do not take account of the diversity of dietary patterns within the population. Maillot et al. [23] modelled healthy diets that met nutrient recommendations at the individual level for the French population by minimising dietary change in order to find a best fit to the person’s food patterns. Their study focused on health as an outcome but did not take into account the environmental impact of dietary intakes. Healthy diets do not necessarily have a lower environmental impact, for example lower GHGE, and therefore it is necessary to consider both health and environmental sustainability together [21, 24]. Modelling diets based on minimising the change to an individual’s current dietary intake allows individual food choices and habits, which are often driven by preferences, cultures or circumstance, to be accommodated. The aim of this study was to determine the range of dietary changes that could be made by adults in a UK population to achieve dietary recommendations for health and to reduce GHGE by minimising, that is making the fewest and easiest, changes to current dietary intakes.

## Methods

### Sample and data

Individual diets were modelled using the reported dietary intakes of each adult in the UK National Diet and Nutrition Survey (NDNS). Using the mathematical optimisation technique of linear programming two new diets were constructed for each person in the sample, one that met only dietary recommendations (referred to as a healthy diet) and one that met dietary recommendations plus a GHGE target (referred to as a sustainable diet), both while minimising dietary changes from their current reported intake. A sustainable diet has multiple dimensions but in the context of this paper it includes only health and reduced GHGE.

#### Current dietary intake data

Dietary data from the NDNS rolling programme (2008-11) were used for the modelling in this study [5]. The NDNS reports food and nutrient intakes for adults (aged 19–94years) based on a 4-day diet diary (*n* = 1491). Food and drink items reported in the diaries are coded and converted to nutrients intakes. For the linear programming 134 food and drinks categories based on NDNS food groups were used, but for reporting these were further grouped into 35 categories, based on the original NDNS food groups and similarity in nutritional content.

#### Greenhouse gas emissions data

Details of the methodology to derive the GHGE data have been described in full previously [20]. In summary, GHGE data were taken from the most comprehensive dataset available in the UK at the time of the study, published by Audsley et al. [25]. The GHGE are for primary commodities (e.g. wheat, sugar, beef) and include emissions from primary production up to the retail distribution centre in the UK (RDC) (*i.e*. they do not include emissions from processing of composite foods, for example wheat to bread, distribution to retail or the home, home storage or cooking and waste disposal). Audsley et al. estimated GHGE values for each commodity based on production in three locations: the UK, the rest of Europe and the rest of the world. In this study to get a single GHGE value for each food, an average was calculated weighting each GHGE value by the proportion domestically produced and imported from Europe and the rest of the world (using UK trade statistics). For composite and processed foods (e.g. biscuits, cheese, pizza) GHGE data were estimated from the ingredients they comprised, using methods published by Wallén et al. [26] and recipes from the sixth edition of McCance and Widdowson’s The Composition of Foods and Supplements [27]. To match the nutrient and GHGE data, the GHGE data were converted to represent the food item as eaten, that is only the edible part of a food item (e.g. excluding banana skins) and adjusting weights for cooking (e.g. rice) using conversion factors from food composition tables [27].

#### Dietary recommendations and greenhouse gas emissions target

The dietary and GHGE constraints (all of which had to be met in the new diets) used to generate new diets in the linear programming are shown in Table 1. Nutrient constraints were based on dietary reference values [2] and food based constraints taken from the UK government dietary guidelines [3]. An upper limit constraint was added to the model for alcohol to comply with recommendations (maximum intakes of 24g/d for men and 16g/d for women [28]). In addition the amount of alcohol in the new diets could not be increased above the individual’s current reported consumption, to avoid recommending increasing alcohol consumption. The fruit and vegetable constraint was based on a minimum of five portions equating to approximately 400g per day [29]. In the linear programming model, estimates for the fruit and vegetables constraint are based on discrete portions, but additional fruit and vegetables found in composite dishes needed to be included (e.g. lasagne, pizza). It is estimated in the NDNS that approximately 25 % of total fruit and vegetable consumption comes from composite dishes [5] and therefore the constraint in the model was set at 300g/d for discrete portions, assuming the remainder comes from composite dishes. Since few people currently achieve the recommendation of 400g/d it was important to include the contribution from composite dishes when minimising the changes people would need to make to their current diet. The constraint for red/processed meat and fish consumption were adjusted to take into account the amounts of these foods in composite dishes. In the NDNS it is estimated that in the categories for red meat and dishes about 57 % is meat, therefore the recommended maximum of 70g/day was adjusted to 121g/day for the meat and meat dishes. Similarly, 54 % of white fish dishes and 73 % of oily fish dishes are estimated to be fish. To meet the recommendation of two portions of fish per week (at least one of which is oily fish) adjusting for composite dishes, the constraint was set as a minimum of 261g/week and 193g/week from white fish and oily fish dishes, respectively. The energy intake reported by individuals in the NDNS was used as the energy constraint in the new diets since the aim of the study was to minimise the modification of current diets and making assumptions about misreporting is problematic.

**Table 1:** Dietary recommendations and greenhouse gas emissions (pre-RDC) reduction targets compared with current dietary intakes of the study population

	GHGE target & dietary recommendations [2, 3]		Current dietary intakes (median)		Proportion meeting recommendations (%)	
	Women	Men	Women	Men	Women	Men
			(*n* = 841)	(*n* = 650)	(*n* = 841)	(*n* = 650]
GHGE (pre-RDC) (kgCO_2_e/d)	≤2.5	≤3.1	2.9	3.6	36.4	34.5
Energy (MJ/d)	8.7	10.9	7.6	9.9	-	-
Protein (g/d)	≥53	≥65	88.4	109.8	90.4	89.2
Total fat (% food energy)	≤35	≤35	29.8	29.8	82.8	84.3
Saturated fat (% food energy)	≤11	≤11	10.7	10.6	54.1	56.9
Carbohydrate (% food energy)	≥50	≥50	49.5	48.1	47.1	39.8
Non milk extrinsic sugars^a^ (% food energy)	≤11	≤11	11.5	12.4	46.0	41.5
Fibre (g/d)	≥18	≥18	12.9	14.6	17.0	29.2
Calcium (mg/d)	≥700	≥700	907	1118	75.9	85.2
Iron (mg/d)	≥14.8	≥8.7	16.6	19.7	57.9	93.7
Zinc (mg/d)	≥7	≥9.5	9.9	12.3	82.0	74.8
Magnesium (mg/d)	≥270	≥300	419.4	567.2	70.9	78.6
Copper (mg/d)	≥1.2	≥1.2	1.6	1.9	68.4	80.5
Sodium (mg/d)	≤2400	≤2400	2776.3	3455.4	37.1	21.5
Selenium (μg/d)	≥60	≥75	55.1	69.2	42.4	39.4
Iodine (μg/d)	≥140	≥140	151.4	194.0	58.4	75.8
Vitamin A (μg/d)	≥600	≥700	751.2	824.2	67.3	60.0
Thiamin (mg/d)	≥0.8	≥1	1.3	1.6	92.2	88.9
Riboflavin.(mg/d)	≥1.1	≥1.3	1.9	2.3	90.4	88.6
Niacin (mg/d)	≥13	≥17	36.2	48.5	93.1	93.8
Vitamin B6 (mg/d)	≥1.2	≥1.4	2.2	2.8	94.3	95.2
Vitamin B12 (μg/d).	≥1.5	≥1.5	5.2	6.4	97.6	99.4
Folate (μg/d)	≥200	≥200	280.6	351.2	83.9	89.1
Vitamin C (mg/d)	≥40	≥40	79.3	86.3	86.2	86.8
Alcohol (g/d)	<16	<24	1.4	8.6	79.8	70.0
Fruit & vegetables (g/d)^a^	≥300	≥300	150.8	141.3	13.2	15.7
White fish & dishes (oily fish & dishes) (g/d)^b^	≥37.3 (27.5)	≥37.3 (27.5)	28.0 (0.0)	25.0 (0.0)	42.2 (15.5)	37.7 (16.3)
Red meat^c^ & dishes (g/d)^b^	<121	<121	133.2	77.3	44.9	68.1

^a^Non milk extrinsic sugars are all sugars added in manufacturing, cooking, or at the table, and those in honey, syrups, fruit juices and fruit purees plus 50 % of the fruit sugars in dried, canned and stewed fruit (similar to free sugars)

^b^dietary recommendations adjusted for the inclusion of composite dishes

^c^beef, lamb, pork

Targets for reducing GHGE tend to be based on national or international emissions rather than for individuals, and compared to a baseline level (e.g. in the UK 1990 emission levels [30]). Currently there is no specific target for the reduction of GHGE within the food system or for individual dietary intakes. The constraint for GHGE used in the model was an estimate of a population based 25 % reduction compared to the 1990 baseline data for UK emissions. Adjusted to be equivalent to pre-RDC GHGE for consistency with the dataset, the targets were calculated as ≤3.1 kgCO_2_e/d and ≤2.5 kgCO_2_e/d, for men and women respectively [31].

### Analysis: linear programming optimisation

Linear programming is a mathematical modelling technique [32] that has been used previously to construct nutritionally complete diets while optimising another objective (e.g. minimising GHGE or monetary cost of the diet). Diets are constructed to minimise an objective, which is a linear function of the food item amounts, i.e.

$$ \mathrm{objective} = {c}_{1\ }{x}_1 + {c}_{2\ }{x}_2 + \dots + {c}_n{x}_n $$

where c_*i*_ is the contribution of a food item *i* to the objective.

In this study the desired improvements in nutrition and reductions in GHGE resulting from proposed dietary changes are built into the linear programming framework as constraints within which the above objective was to be minimised. The dietary constraints comprised either a lower limit (i.e. protein, fibre, carbohydrates, vitamins, minerals, fruit and vegetables, and fish) or an upper limit (i.e. sodium, total fat, saturated fatty acids, non-milk extrinsic sugars (NMES) and alcohol) (see Table 1). Most were expressed in absolute amounts but fats, carbohydrate and sugar were expressed as percentages of total energy. The *m* macronutrient and micronutrient requirement limits were denoted as *b*_1_, *b*_2_, …, *b*_*m*_, and with each food group *i* contributing *a*_*ij*_ per unit weight to requirement *j*, a set of *j* dietary constraints were established, such as:

$$ {a}_{j1}{x}_1 + {a}_{j2}{x}_2+\dots +{a}_{jn}{x}_n\ \ge\ {b}_j $$

The energy required exact equality to the value specified that matched the reported energy intake for each individual. A GHGE limit was imposed by adding an extra constraint where the *a*_*ij*_ was the GHGE associated with each different foods.

Lower and upper limits l_*i*_ and u_*i*_ on individual food items were set as additional constraints, (i. e. *l*_*i*_ ≤ *x*_*i*_ ≤ *u*_*i*_) to minimise change. These were derived from restricting the change in weight of any food item in the current diet, first up to maximum of ≤50 % but where this was insufficient to achieve the dietary and GHGE constraints it was then increased to a maximum of ±100 %. Thus the model preferentially, where possible, met the dietary and GHGE constraints with foods already eaten rather than introduce new foods or remove foods. Nutrient composition data *a*_*ij*_ for all the food items came from the NDNS.

Linear programming was carried out by using the GNU Linear Programming Kit as implemented in the lpSolveAPI package of the R [3.10] statistical software environment (R Foundation for Statistical Computing, Vienna, Austria).

### Improving diets with minimal change

The primary purpose of this study was to investigate how much change to current diets is are needed in order to construct a healthy diet and a sustainable diet. Using individual diets reported in the NDNS the number of people who already met all dietary recommendations was established, and then the number who met both the dietary recommendations and the GHGE target. For those not meeting the recommendations it was assumed that dietary modification was more achievable by minimising changes to the food and drinks they habitually ate. A stepwise approach was used to modify each of the diets using the rationale that each step required a greater and more difficult change to dietary habits, as follows; 1. no change to the foods eaten only the quantity, 2. addition of new foods, 3. addition of new foods and a greater change to the quantity of foods already being eaten and 4. removal of foods from the diet. Modifying the amount of any food currently eaten was seen to have the least impact on changing dietary habits. The second step was to add new foods because people tend to be more willing to accept adding new foods to their diet than removing existing foods [33], which is associated with behavioural economic theories of loss aversion [34]. The amounts of foods reduced or increased, or added or removed was determined in the modelling, which selects the diet that meets all the constraints and requires the smallest amount of change from the many possible diets that achieved the goal. This approach was applied to each individual diet of the sample.

#### Step 1 No change to the foods being eaten, only the quantity

The amount of any food item currently eaten was changed (increased or decreased) by up to *P* %. Thus if *z*_i_ was the amount of a food item currently eaten, the requirement for the amount *x*_*i*_ was set in the new diet to be:

$$ \left(1-\frac{P}{100}\right){z}_i\ \le\ {x}_i\ \le\ \left(1+\frac{P}{100}\right){z}_i $$

P was gradually increased from 1 to 50 % in increments of 1 % until a new diet that satisfied the constraints was identified. When a value of P was found to meet all constraints, the solution space was very small (since for P-1 it did not exist) and the objective optimised had negligible effect. Therefore a constant vector c_i_ = 1 (*i* = 1,…,*n*) was used.

#### Step 2 Changes to the amount of any food already being eaten and addition of new foods

For people where a 50 % change in the quantity of any food currently eaten was not enough to meet the constraints, the next step was to add new items to the diet. Since the aim was to minimise change, c_i_ was set equal to 1 for food items not currently consumed and zero otherwise, with *P* fixed at 50 %. Thus the weight of the new food items added to the diet was minimised meaning adding as little as possible to the diet. In the case of those who identified themselves as vegetarians (*n* = 29) no meat or fish items could be added to the diet.

#### Step 3 Greater reduction in the amount of any food already eaten and new foods added

When people could not meet the constraints with steps 1 and 2 the maximum reduction in the quantity of any food in the current diet was raised to 75 %, but the increase was still restricted to a maximum of 50 %, and new foods could be added to the diet.

#### Step 4 Removal of any food from the current diet

For those who could not meet the constraints after steps 1 to 3, foods were removed from the current diet in order to achieve all the constraints by setting the maximum reduction to 100 %.

Individual models for each of the 1491 diets were run twice; first where only the dietary constraints had to be met (healthy diet), and second where both the dietary and GHGE constraints had to be met (sustainable diet). In the first model it was possible that a modified diet could result in an increase in GHGE in order to satisfy the dietary constraints. An increase in GHGE was also possible in the second model because to minimise change and meet dietary constraints a small increase in GHGE could occur in the case where the GHGE of the current diet was below the GHGE maximum constraint.

## Results

Table 1 shows the current dietary intakes reported by men and women in the NDNS and the number of people achieving each of the dietary recommendations. Dietary fibre and sodium was the nutrient recommendations that the fewest people met, with only 17 % of women meeting the target for fibre and 22 % of men consuming no more than the recommended maximum intake of sodium. Approximately 90 % of the population met protein requirements and the majority were achieving requirements for vitamins and minerals, with the exception of selenium.

### Minimising dietary change

Table 2 shows the stepwise dietary changes for the sample to achieve healthy diets and sustainable diets. Only one person in the sample of 1491 met all the dietary recommendations in their current diet. Typically greater changes were needed to achieve a sustainable diet than simply a healthy diet. Only 7.5 and 4.6 % of the sample could achieve a healthy diet and a sustainable diet, respectively, by increasing or decreasing, by up to 50 %, the amount of any food or drink that they already ate. Women were more likely than men to achieve the new dietary recommendations with this degree of change (healthy diets: 10.0 *vs* 3.2 %, sustainable diets: 6.0 *vs* 2.6 %). In the whole sample 59 and 53 % of people could achieve a healthy diet and a sustainable diet, respectively, at step 2 (i.e. up to 50 % change in any food eaten plus new foods added). A median of five new foods were added to achieve either of the new diets, with oily fish being the most common food added. The quantities of any new food added were not excessive or unrealistic. For only a small minority (3.9 and 4.8 % respectively) it was necessary to remove foods (step 4), and for these people a median of seven and eight foods were removed to achieve a healthy diet and a sustainable diet, respectively.

**Table 2:** Stepwise dietary changes to achieve healthy diets and sustainable diets

	Healthy diets			Sustainable diets		
	cumulative percent	GHGE (kgCO_2_e/d)^a^		cumulative percent	GHGE (kgCO_2_e/d)^a^	
	(*n* = 1491)	mean (SD)		(*n* = 1491)	mean (SD)	
		current	new		current	new
No change	0.1	3.24	3.24	0.0	-	-
*Step 1*: *up to a maximum of 50* % *change in the amount of any food group currently eaten*						
1–20 % change	0.6	2.59 (0.71)	2.67 (0.65)	0.2	2.07 (0.65)	2.14 (0.60)
21–50 % change	7.5	3.24 (0.90)	2.99 (0.65)	4.6	2.78 (0.66)	2.46 (0.36)
*Step 2*: *up to a maximum of 50* % *change in the amount of any food group currently eaten and new foods added to the diet*						
1–3 new items	20.6	3.12 (1.00)	2.96 (0.76)	16.2	2.85 (0.72)	2.47 (0.35)
4–6 new items	45.3	3.14 (1.14)	2.75 (0.73)	38.7	2.93 (0.90)	2.44 (0.44)
7–13 new items	58.6	2.64 (1.12)	2.51 (0.65)	52.9	2.64 (1.06)	2.37 (0.46)
*Step 3*:*up to a maximum 50* % *increase and*/*or 75* % *reduction in amount of any food group currently eaten and new foods added to the diet*						
1–3 new items	67.8	4.30 (1.71)	3.38 (0.92)	61.9	3.99 (1.25)	2.66 (0.36)
4–6 new items	86.3	4.09 (1.71)	3.13 (0.81)	83.2	4.14 (1.51)	2.67 (0.40)
7–10 new items	96.1	3.37 (1.74)	2.69 (0.73)	95.2	3.87 (2.01)	2.48 (0.45)
*Step 4*: *up to a maximum of 50* % *change in the amount of any food group currently eaten and food items removed from the diet*						
1–24 items removed	100	4.80 (2.98)	3.46 (1.02)	100	5.25 (3.08)	2.70 (0.46)
Total change in GHGE (kgCO_2_e/d)		3.44 (1.58)	2.91 (0.81)		3.44 (1.57)	2.52 (0.43)

^a^GHGE data are from production to the regional distribution centre (pre-RDC), not the full life cycle

Difficulties in achieving a healthy diet tended to be dominated by specific nutrients, particularly sodium. If the sodium recommendation was dropped as a constraint the proportion of the sample able to meet all requirements with up to 50 % change (step 1) increased from 8 to 19 % for a healthy diet.

Thirty five percent of the sample met the target set for GHGE in their current diet and after modelling the healthy diet this increased to 47 %. As seen in Table 2, for the healthy diet there is a small but gradual shift towards lower GHGE as more changes were made to the diets. The mean GHGE reduced by only 15 % for the healthy diets compared with a reduction of 27 % for the sustainable diets, but this required greater dietary changes.

#### Dietary changes for healthy diets and sustainable diets

**Fig. 1:**
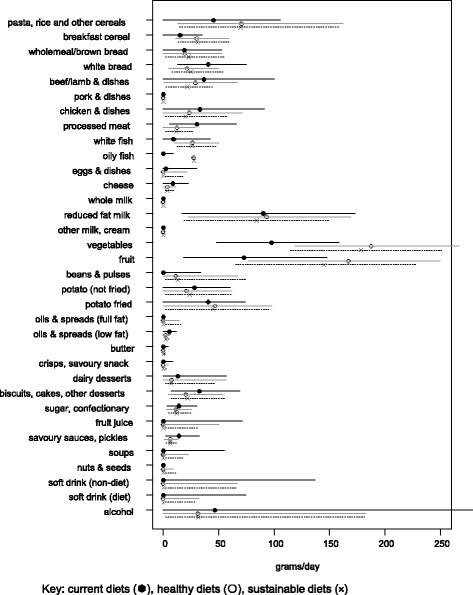
Median intake (±IQR) of each food group in the current and new diets

Figure 1 shows the median intakes of food and drinks across the population for the two new diets compared with current dietary intakes. For both of the new diets the foods most frequently increased in quantity among current consumers were vegetables, fruit, potatoes, nuts & seeds, breakfast cereals and cereals (e.g. pasta and rice) and reductions in sweet foods (biscuits, cakes and desserts), processed meats, alcohol and white bread. Fruit and vegetables tended to increase more in the healthy diet than sustainable diet, and total meat consumption, especially beef and lamb, decreased more to achieve sustainable diet than just a healthy diet.

**Fig. 2:**
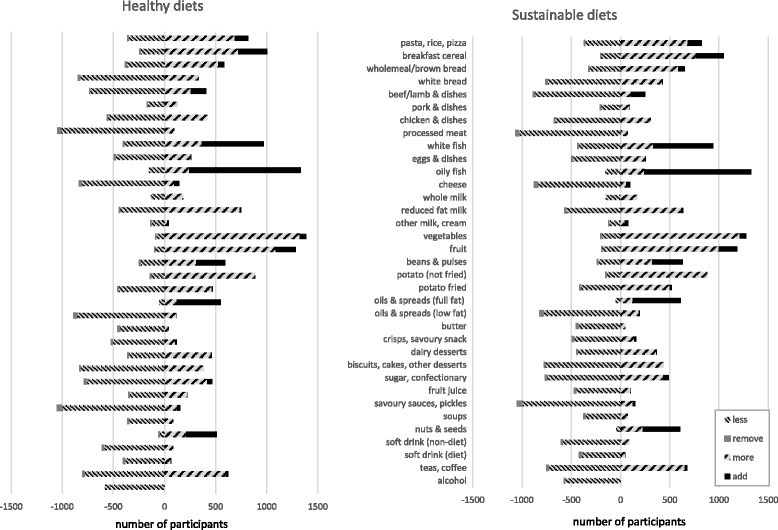
Number of individuals needing to eat less, more, remove or add foods to their diet

The dietary changes varied depending on the original diet, with the same food being increased in quantity for some people, reduced the quantity for others, added as a new food or for a minority removed from their diet (see Fig. 2). The foods most commonly added were fish, beans, nuts and seeds, and oils and spreads, the latter two only in very small quantities as shown in Fig. 1. The foods not added to any of the new diets included alcohol, sweet foods, non-diet soft drinks, some meats (chicken, processed meats), butter, whole milk, and potatoes. The reduction in fruit and vegetables for a minority of people occurred because they were consuming more than the minimum requirements and reducing the amount helped lower the GHGE of the diet. Similarly, foods such as oils and spreads tended to be increased in the new diets in small quantities because they provided energy without increasing the sodium content of the diets, which was one of the hardest nutrient recommendations to meet.

## Discussion

This study used a modelling approach to determine the minimum change individuals would need to make to their current diets to meet the criteria for a healthy diet and a sustainable diet. Only a small number of people could meet the dietary recommendations by simply changing the quantities of any food or drink they currently consumed by up to 50 %. While the majority had to make more substantive dietary changes to the amount of any food currently eaten and addition of new foods to their diet, for only a very small proportion of the sample foods needed to be removed from their diet. Taking an individual approach to dietary change and considering the whole diet rather than single nutrients illustrates the complexity and many different ways diets can be optimised to meet dietary recommendations and reduce GHGE. For the majority of food items, some people increased the quantity and others reduced the quantity, while for others the food was added or removed. This variability would not be captured in modelling the average diet of the population or the range of different dietary changes that would achieve the same outcome. Shifting to a healthy diet alone only achieved a small reduction in GHGE at a population level and the addition of a specific GHGE constraint was needed to reduce GHGE by a substantial amount.

The amount and type of dietary changes needed varied between individuals. In many cases it was a single nutrient or food constraint that made a feasible solution impossible. If, for example, a diet had more than double the maximum recommended level of a nutrient with an upper limit (e.g. sodium or saturated fat) no new diet with only 50 % change could achieve the recommendation. Conflicts between nutrient recommendations often meant that no feasible solution existed. Sodium and fibre, for example, were found to be the most problematic nutrients. For these nutrients recommendations are in opposing directions but they often co-exist in foods (e.g. bread, breakfast cereals). Sodium has been highlighted in previous studies as a problem nutrient when trying to simultaneously achieve multiple dietary recommendations [35]. In the NDNS table salt added to food is not included but if this was included it would make the sodium target even harder for some people to achieve. Difficulties in trying to achieve multiple dietary targets for a healthy diet highlight the limitations of taking a reductionist approach and studying nutrients in isolation, rather than considering the whole diet.

Similarly categorising food as good or bad in terms of perceived healthiness or environmental impact is recognised as an oversimplification. The data in Fig. 2 shows that most of the food and drink items were not exclusively increased or decreased in moving people towards a healthy or sustainable diet. There was no increase or addition of alcohol, but this was forced by specifying in the model that no increase was permitted in the new diet because it was deemed inappropriate to recommend anyone to drink more alcohol than they currently consume. Without this constraint alcohol would increase for some people because it contributed to meeting energy requirements without increasing upper limited nutrients, such as sodium, fat or NMES, and has lower GHGE than other foods such as meat and dairy products. Meat is typically highlighted as a food that should be reduced for climate change mitigation and to limit environmentally damage [10, 15], but as seen in the results of this study, for a small number of people meat was increased to optimise the nutritional composition of the diet. In limited quantities unprocessed meat can provide a good source of nutrients. It is also a food that many people are reluctant to reduce or remove from their diet, even when they are made aware of the environmental damage of meat production [36, 37]. Reluctance to eat less meat is for a variety of personal, social and cultural reasons that need to be recognised, and generally eating less meat has limited social and political appeal. Similarly high fat and high sugar foods are often eaten for sensory pleasure and therefore it would be difficult and unrealistic to suggest removing them from current dietary intakes [38]. As demonstrated in this study, all these foods can be eaten in varying quantities as part of a healthy diet, for example where meat is an important part of someone’s diet it could be retained and other foods limited, which could give the same net health and environmental outcome. Differences in personal food choices, preferences and other factors influencing dietary intakes, as well as differences between socio-economic groups, can be taken into consideration by using this approach.

Improving dietary intakes of the population has proved extremely difficult, and therefore understanding current dietary habits and adopting the approach of minimising changes people have to made could potentially make it more realistic and achievable. While this has been done at an individual level in this study, the same methodology can be applied at a population level to tailor advice to targeted groups, for example different age, sex, income or ethnic groups. In this study the stepped approach used was based on starting with what we considered easier changes, where the foods already eaten were not changed only the amount of them eaten. This equates to changing portion sizes or frequency of eating these items. When this was not sufficient to meet the recommendations new foods were added to the diet and only when this still failed to meet recommendations were foods removed from the current diet. It has been reported that people are more willing to add new foods to their diets than remove foods they currently eat [33]. Dietary guidelines focus on objective goals for health to prevent disease and deficiencies, and more recently on reducing the environmental damage, but they do not tend to consider the social, personal, economic and cultural aspects of eating [18, 39]. Eating habits are socially constructed and food is important to our sense of identity and it has been recognised for a long time that for many people this matters more than nutrition, but it is still tends to be overlooked [40, 41]. Previous research shows that in some circumstances when people make changes to eat a healthier diet that they can experience feelings of social exclusion among their peers [42, 43]. This needs to be factored into dietary advice, following the lead by the Brazilian government who in their new dietary guidelines have incorporated some of the important social and cultural aspects of eating habits in their population [19].

In the assessment of reported dietary intakes misreporting is widely recognised as a problem and this is no exception in the NDNS study [44]. Misreporting tends to be biased towards under-reporting, with reported energy and nutrient intakes being lower than habitual intakes [45]. Basing the energy requirement for the new diets on reported energy intakes is likely to overestimate the changes required to achieve a nutrient dense diet, but underestimate the change needed for nutrients with maximum recommendations (e.g. sodium). The UK’s reference nutrient intake values are population based recommendations and are set at levels to ensure that the needs of 97.5 % of the population are met [2], and it would not be expected that all individuals in a group of 1491 people would meet each of the dietary recommendations. By exceeding this requirement in the population, we may have overestimated the amount of change needed to the current diets. Furthermore, dietary intakes were recorded for 4 days and some may not be representative given the day-to-day variations in dietary intakes. Some food items eaten infrequently could be missed, for example the recommendation for oily fish for one portion per week and this could be missed in a 4 day diet diary. The stepwise approach used in the analysis to minimising change to the diet was based on previous studies and behavioural theories that people are habitual in what they eat and would rather have gains than losses [33, 34]. However, there are various ways to minimise change and this may not be the preferred order of change for everyone, but information about individual’s preferences was not available in this sample. The monetary cost of the dietary changes was not included in this study, but this is as an important consideration since an increase in cost of the diet could be a deterrent, particularly for those with lower incomes.

The GHGE data were from the most comprehensive dataset for UK food available at the time of the study, but the data are limited as they do not cover emissions over the full lifecycle. Emissions associated with processing to composite products (e.g. cakes, pizza), home use (e.g. cooking or refrigeration) and waste disposal are not included. The aim of the study was to reduce GHGE, but this is only one of many indicators of an environmentally sustainable diet. The dietary recommendation to increase consumption of fish, for example, does not take into account the conflict between demand and the sustainability of fish stocks [46].

A strength of this study was in using an approach to minimise dietary change that incorporates individual food choices and thereby adding a social, personal and cultural dimension that is often lacking in recommending dietary changes. The study advances previously published research as it considers not only the changes required to shift towards healthy diets [23, 47, 48], but the changes needed to achieve healthy diets with lower GHGE. It showed that while some of the dietary changes are similar between the two new diets, a sustainable diet typically requires more changes, for example, a greater reduction in meat and vegetables, than for a healthy diet.

## Conclusions

This study shows the diverse changes that can be made to current dietary intakes across the population to achieve healthy diets and sustainable diets. Focusing only on healthy diets does not substantially reduce the average GHGE of the diet, which supports the need to develop new dietary guidelines that incorporate recommendations that will reduce environmental impacts. There needs to be consideration of the diversity of diets and dietary behaviours in the population that are being driven by different social, personal and cultural factors. This has illustrated the range of possible routes to achieve healthy and sustainable diets, and taking into account dietary habits may be a more effective approach to improving dietary intakes. The same approach could be taken to develop more appropriate dietary advice for different subgroups of the population.
